# A disulfidptosis-related lncRNAs cluster to forecast the prognosis and immune landscapes of ovarian cancer

**DOI:** 10.3389/fgene.2024.1397011

**Published:** 2024-07-09

**Authors:** Jiahui Wei, Ming Wang, Yumei Wu

**Affiliations:** Beijing Obstetrics and Gynecology Hospital, Beijing Maternal and Child Health Care Hospital, Capital Medical University, Beijing, China

**Keywords:** ovarian cancer, disulfidptosis, lncRNA, prognosis, immune

## Abstract

**Objective:**

Disulfidptosis is a newly recognized form of regulated cell death that has been linked to cancer progression and prognosis. Despite this association, the prognostic significance, immunological characteristics and treatment response of disulfidptosis-related lncRNAs (DRLs) in ovarian cancer have not yet been elucidated.

**Methods:**

The lncRNA data and clinical information for ovarian cancer and normal samples were obtained from the UCSC XENA. Differential expression analysis and Pearson analysis were utilized to identify core DRLs, followed by LASSO algorithm. Random Survival Forest was used to construct a prognostic model. The relationships between risk scores, RNA methylation, immune cell infiltration, mutation, responses to immunotherapy and drug sensitivity analysis were further examined. Additionally, qRT-PCR experiments were conducted to validate the expression of the core DRLs in human ovarian cancer cells and normal ovarian cells and the scRNA-seq data of the core DRLs were obtained from the GEO dataset, available in the TISCH database.

**Results:**

A total of 8 core DRLs were obtained to construct a prognostic model for ovarian cancer, categorizing all patients into low-risk and high-risk groups using an optimal cutoff value. The AUC values for 1-year, 3-year and 5-year OS in the TCGA cohort were 0.785, 0.810 and 0.863 respectively, proving a strong predictive capability of the model. The model revealed the high-risk group patients exhibited lower overall survival rates, higher TIDE scores and lower TMB levels compared to the low-risk group. Variations in immune cell infiltration and responses to therapeutic drugs were observed between the high-risk and low-risk groups. Besides, our study verified the correlations between the DRLs and RNA methylation. Additionally, qRT-PCR experiments and single-cell RNA sequencing data analysis were conducted to confirm the significance of the core DRLs at both cellular and scRNA-seq levels.

**Conclusion:**

We constructed a reliable and novel prognostic model with a DRLs cluster for ovarian cancer, providing a foundation for further researches in the management of this disease.

## Introduction

Ovarian cancer is recognized as the most lethal gynecological cancer, with 90% of cases being epithelial ovarian cancers. These are typically diagnosed at an advanced stage and carry a poor prognosis (Armstrong et al., 2021; Chen Z et al., 2023). Global statistics from 2020 reported 313,959 new cases of ovarian cancer and 207,252 new deaths worldwide (Bukłaho et al., 2023). In 2024, 19,680 new cases and 12,740 new deaths of ovarian cancer are projected to occur in the United States (Siegel et al., 2024). It is projected that the number of women affected by ovarian cancer will surpass 445,000 by 2040 (Zoń and Bednarek, 2023). Due to the atypical clinical manifestations of early-stage ovarian cancer, over 60% of patients are diagnosed at an advanced stage (Yang S. et al., 2023). Additionally, platinum-resistant recurrence is also a major contributing factor to the poor prognosis and high mortality rates associated with ovarian cancer (Shoji et al., 2022). The 5-year survival rate for ovarian cancer is less than 50%, with patients in advanced stages having a survival rate of about 20%–30% (Jazwinska et al., 2023; Liu et al., 2023a; Liu et al., 2023b). Therefore, it is crucial to select novel and efficient prognostic biomarkers for ovarian cancer patients.

In the era of precision oncology, there is a growing focus on the molecular characteristics and heterogeneity involved in tumor development (Yang Z. et al., 2023). Multiple studies have shown that cancer cells frequently undergo metabolic reprogramming to facilitate their rapid growth and combat the oxidative stress caused by metabolic disruptions during tumor progression and spread (Zheng et al., 2023). Disulfidptosis represents a newly identified form of controlled cell death triggered by disulfide stress (Zhang D. et al., 2023). SLC7A11, a cystine transporter commonly upregulated in cancer cells, has been found to increase reliance on glucose for energy production (Xia et al., 2023). In situations where glucose is limited and SLC7A11 levels are high, depletion of NADPH can result in disulfide bond stress, causing abnormal accumulation of disulfide bonds in actin, alterations in protein function, and ultimately cell death (Liu et al., 2024). This significant discovery is anticipated to improve the identification of new prognostic markers (Xue et al., 2023). The involvement of disulfidptosis in tumorigenesis across various cancer types suggests its potential as a diagnostic and therapeutic indicator (Machesky, 2023). Xu et al. (Xu et al., 2024) demonstrated that disulfidptosis-related lncRNA could act as a prognostic biomarker and therapeutic target for hepatocellular carcinoma. Additionally, Xiao et al. (Xiao et al., 2024) created a prognostic signature linked todisulfidptosis prolapse that correlated with response to immunotherapy in colorectal cancer. Xie et al. (Xie et al., 2024) quantified the disulfidptosis activity score in pan-cancer cells and found that lower grade glioma had the highest average score, while lymphoblastic acute myeloid leukemia had the lowest disulfidptosis activity score. They also observed that mutations in disulfide-related genes were consistently present in cervical cancer samples, but no mutations were detected in ocular melanomas. However, there is currently a lack of information on disulfidptosis specifically in ovarian cancer.

Long non-coding RNAs (lncRNAs) are transcripts that exceed 200 nucleotides in length and do not encode proteins They are involved in chromatin remodeling, transcriptional and post-transcriptional regulation, and have significant implications in the development of different types of cancers (Fang and Fullwood, 2016). Previous studies have reported that lncRNAs play crucial roles in various biological processes such as cell migration, invasion, proliferation, and apoptosis (Xia et al., 2023). Dysregulated expression of lncRNAs has been observed in different cancer types, suggesting their significance in tumorigenesis (Liu Y. et al., 2023). Variations in disulfidptosis activity scores have been noted among different cell types, including malignant cells, myeloid cells, parietal cells, endothelial cells, and cancer-associated fibroblasts (Xie et al., 2024). There is currently no literature on the relationship between disulfidptosis-related lncRNAs (DRLs) and ovarian cancer. This study aimed to develop a prognostic model for ovarian cancer patients using DRLs and conducted immune-related analyses to provide new insights for ovarian cancer research.

## Materials and methods

### Data collection

The lncRNA data and related clinical information of 418 TCGA-ovarian cancer specimens and 88 GTEx-normal samples were obtained from the UCSC XENA (https://xenabrowser.net/datapages/), which have been removed from batch effects (Vivian et al., 2017). The gene expression profile information and clinical data are easily accessible and publicly available, eliminating any ethical concerns. GTF files (GRCH38) were obtained from The Encyclopedia of DNA Elements (https://www.gencodegenes.org/#).

### Differential expressed analysis and the core DRLs acquirement

Differential expression lncRNAs (DELs) were identified using FDR <0.05 and |log2fold change (FC) > 1| as the criteria (Xia et al., 2023). The analysis of DELs between ovarian cancer patients and normal samples was conducted using the R package ‘limma’. A total of 15 disulfidptosis-related genes (FLNA, FLNB, MYH9, TLN1, ACTB, MYL6, MYH10, CAPZB, DSTN, IQGAP1, ACTN4, PDLIM1, CD2AP, INF2, SLC7A11) were compiled from previously published studies (Wang T. et al., 2023). The correlation between DELs and disulfidptosis-related genes was then evaluated through Pearson analysis., with a significant correlation defined as having an absolute correlation coefficient (|R|) ≥ 0.3 and *p* < 0.001. Besides, the least absolute shrinkage and selection operator (LASSO) regression with 5-fold cross-validation was utilized to identify the core DRLs.

### Construction of a prognostic model and survival analysis

Random Survival Forest (RSF) is a machine learning method that extends the random forest method to survival data. RSF captures the interplay of nonlinear effects and variables, computes the cumulative risk function for each sample, and aggregates it by survival time to predict comprehensive mortality outcomes, making it well-suited for survival data analysis (Wang et al.,202b). In this study, RSF was used to analyze the core DRLs and develop a prognostic model for ovarian cancer patients. The ‘predict function’ in R was employed to calculate risk scores for each patient. In this study, we utilized the surv_cutpoint function from the R package to determine the optimal cutoff value for 8 core DRLs in predicting the prognostic risk of ovarian cancer. Patients were then categorized into low-risk and high-risk groups based on this cutoff value, and the stability of these groups was assessed using principal component analysis (PCA). Finally, survival curves between the two groups were compared.

### Functional enrichment analysis and RNA methylation associated with the core DRLs

To investigate the biological functions and pathways associated with DRLs, Gene Set Enrichment Analysis (GSEA) was utilized for assessing differences in enrichment (Zhang C. et al., 2023). RNA methylation can impact RNA processing, translation, and degradation, thereby regulating cellular processes such as cell self-renewal, apoptosis, differentiation, tumorigenesis, and immune cell infiltration in the tumor microenvironment, thereby influencing the physiological and pathological processes of cancer cells (Chen J et al., 2023). A total of 23 m6A modification genes, 12 m5C modification genes, and 10 m1A modification genes were identified from the literature (Xia et al., 2023). The correlation between the core DRLs and RNA methylation genes was then calculated to explore their potential relationship.

### Immune infiltration analysis

The study utilized the CIBERSORT algorithm to analyze the composition of tumor-infiltrating immune cells and compare the differences in immune cell proportions between high-risk and low-risk groups (Newman et al., 2015). Additionally, the ESTIMATE algorithm was employed to assess differences in immune, stromal, and tumor purity scores between the two risk groups (Liao et al., 2023). Single-Sample Gene Set Enrichment Analysis (ssGSEA) in the GSVA R package was used to quantify the infiltration levels and functions of 28 immune cell types based on established gene signatures (Hänzelmann et al., 2013; Xia et al., 2023). Moreover, the Tumor Immune Dysfunction and Exclusion (TIDE, https://tide.dfci.harvard.edu/) was utilized to predict the response to immunotherapy by evaluating tumor immune dysfunction and evasion (Wang et al., 2022).

### Mutation and drug sensitivity analysis

Tumor mutation burden (TMB) is quantified as the number of somatic nonsynonymous mutations, or all mutations, per megabase in the gene region identified through whole exome sequencing or targeted sequencing in tumor samples (Liang et al., 2023). Waterfall plots were constructed using the ‘maftools’ package in R to determine the frequency of point mutations in samples and examine the relationship between TMB and risk scores (Liu S. et al., 2023). The effectiveness of targeted therapy was forecasted using the ‘pRRophetic’ package (Liu et al., 2022). Sensitivity to different drugs was assessed using the semi-maximum inhibitory concentration index (IC50) to explore the therapeutic advantages for ovarian cancer patients (Liu S. et al., 2023).

### Cell culture

Human ovarian cancer cell line (A2780) and normal ovarian cell line (IOSE80) were purchased from BeNa Culture Collection (Henan, China), and cultured in RPMI 1640 medium supplemented with 10% of fetal bovine serum, 100 U/mL of penicillin, and 100 μg/mL of streptomycin (Procell, Wuhan, China). Human ovarian cancer cell line (OVCAR-3) was purchased from Wuhan Procell Life Science & Technology Co., Ltd. (Wuhan, China), and cultured in RPMI 1640 medium supplemented with 0.01 mg/mL insulin, 20% of fetal bovine serum, 1% penicillin/streptomycin (Procell, Wuhan, China). Cells were cultured at 37°C in a humidified incubator with 5% CO2. Prior to experimentation, all cell lines underwent testing for *mycoplasma* contamination and were identified using short tandem repeat analysis.

### RNA extraction and quantitative real-time PCR analysis

Total RNA Extraction Kit (Beijing Solebo Technology, Beijing, China) were used to extract RNA. cDNAs were synthesized using the RevertAid RT kit (Thermo Fisher Scientific, Beijing, China). RT-PCR was performed using the SYBR green assay (Beijing Qihangxing Biotechnology, Beijing, China) on an AB 7500 machine (Applied Biosystems Inc., USA). The SYBR primers used in this study were listed (Supplementary Table S1). GAPDH served as an internal control for normalization. Relative RNA abundance (fold change) of each lncRNA was calculated using the standard 2^−ΔΔCT^. Each sample was examined in triplicate.

### Single-cell RNA sequencing data analysis

To further investigate the expression of the 8 core DRLs cluster at the single-cell RNA sequencing (scRNA-seq) levels, scRNA-seq data from ovarian cancer tissues were obtained from the GEO dataset GSE115007 (Tang-Huau et al., 2018), available in the public Tumor Immune Single-cell Hub (TISCH) database (http://tisch.comp-genomics.org/home/) (Sun et al., 2021). The quality control standards included 500 cells per data set, 1000 UMI counts per cell, and 800 genes per cell. The standards of quality control are cell number per dataset >1,000, UMl count per cell >1,000, and gene number per cell >500. For each collected dataset, a uniform analysis pipeline -- MAESTRO was adopted to perform quality control, clustering and cell-type annotation (Liu et al., 2023e). The scRNA-seq was conducted by the platform of 10×Genomics.

### Statistical analyses

All statistical analyses were conducted using the statistical programming language R. The RSF model was built using the ‘rfsrc’ function from the ‘randomForestSRC’ R package (He et al., 2024). Kaplan-Meier (KM) analysis and the area under the curve (AUC) of the time-dependent receiver operating characteristic (ROC) were employed to compare the 1-year, 3-year, and 5-year survival prognoses as well as prognostic risk performance between the two groups (He et al., 2023). GraphPad Prism V.8 was utilized for qRT-PCR analysis and graphing. In all analyses, *p* < 0.05 was used to indicate statistical differences.

## Results

### Features selection of the core DRLs

**FIGURE 1:**
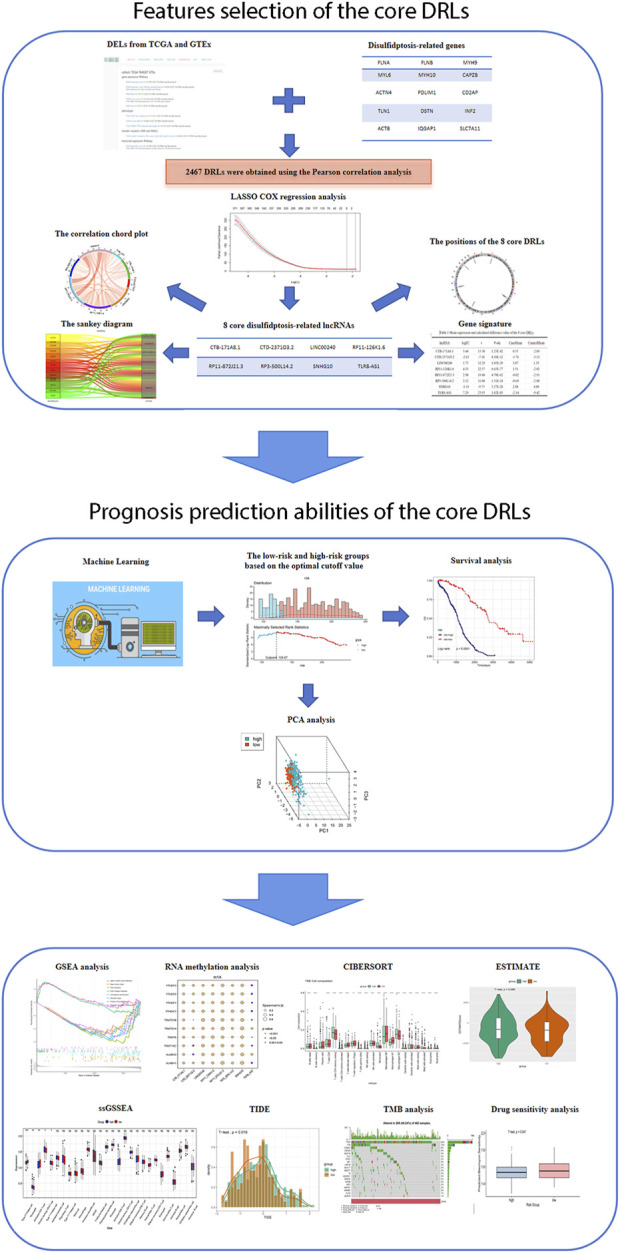
Flowchart of study design.

**FIGURE 2:**
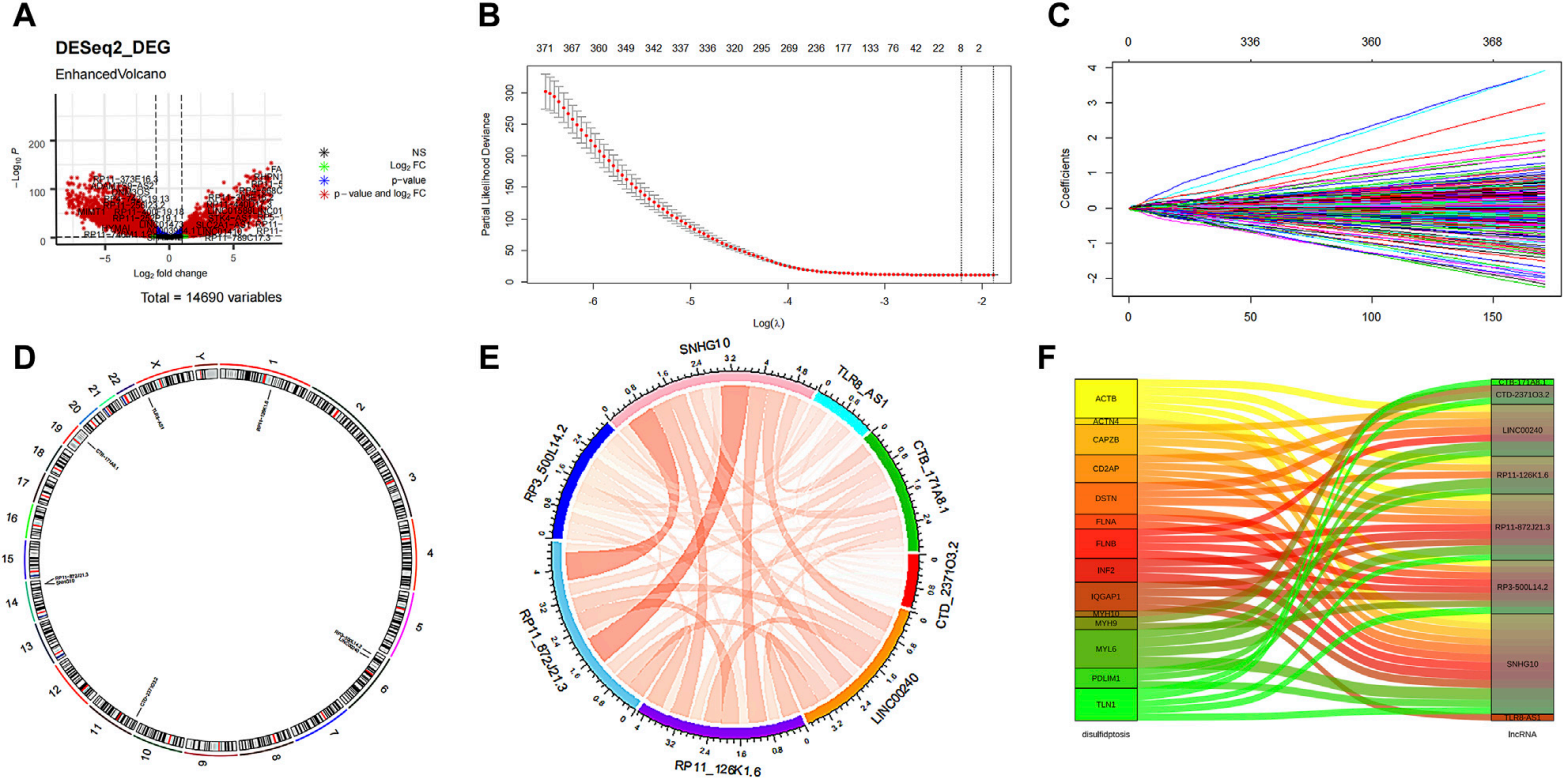
Features selection of the core DRLs. **(A)** Volcano plot of the differential expression lncRNAs. The red dots represented differentially expressed lncRNAs. **(B)** The minimum criteria and **(C)** coefficients were counted by the LASSO Cox regression with 5-fold cross-validation to construct a disulfidptosis-related lncRNAs (DRLs) cluster to forecast the prognosis of ovarian cancer patients. **(D)** The positions of the 8 core DRLs on the chromosome. **(E)** The correlation chord plot showed the correlation of the 8 core DRLs. Red represented positive correlation. The darker the color and the thicker the line represented the higher the correlation strength. **(F)** The sankey diagram demonstrated the relation between the 8 core DRLs and disulfidptosis-related genes.

The flowchart illustrating the data collection, categorization, and analysis process is presented in Figure 1. A total of 7,174 DELs were identified in ovarian cancer patients compared to normal samples, with 3,438 upregulated and 3,736 downregulated (Figure 2A). Subsequently, 2,467 DRLs were identified through Pearson correlation analysis. The LASSO regression was utilized to identify 8 core DRLs (CTB-171A8.1, CTD-2371O3.2, LINC00240, RP11-126K1.6, RP11-872J21.3, RP3-500L14.2, SNHG10, and TLR8-AS1) (Figs. 2B, 2C). The mean expression levels and calculated difference of these 8 core DRLs were summarized in Table 1. The positions of the 8 core DRLs on the chromosome were depicted in Figure 2D. Furthermore, a correlation chord plot was used to visualize the relationships among the 8 core DRLs (Figure 2E). Additionally, a sankey diagram was employed to illustrate the connections between the 8 core DRLs and disulfidptosis-related genes (Figure 2F).

**TABLE 1:** Mean expression and calculated difference value of the 8 core DRLs.

lncRNA	logFC	t	P.adj	CaseMean	ControlMean
CTB-171A8.1	3.44	15.30	1.25E-42	0.55	−2.89
CTD-2371O3.2	−2.63	−7.18	8.50E-12	−5.76	−3.13
LINC00240	1.73	12.29	1.05E-29	3.07	1.33
RP11-126K1.6	4.33	22.57	6.65E-77	1.51	−2.82
RP11-872J21.3	2.90	19.48	4.78E-62	−0.02	−2.91
RP3-500L14.2	2.12	11.00	1.31E-24	−0.69	−2.80
SNHG10	−1.19	−9.75	5.27E-20	2.88	4.08
TLR8-AS1	7.29	23.95	1.62E-83	−2.14	−9.42

CaseMean: The average expression value of the case group. ControlMean: The average expression value of the control group.

### Evaluation of the 8 core DRLs at the cellular level

**FIGURE 3:**
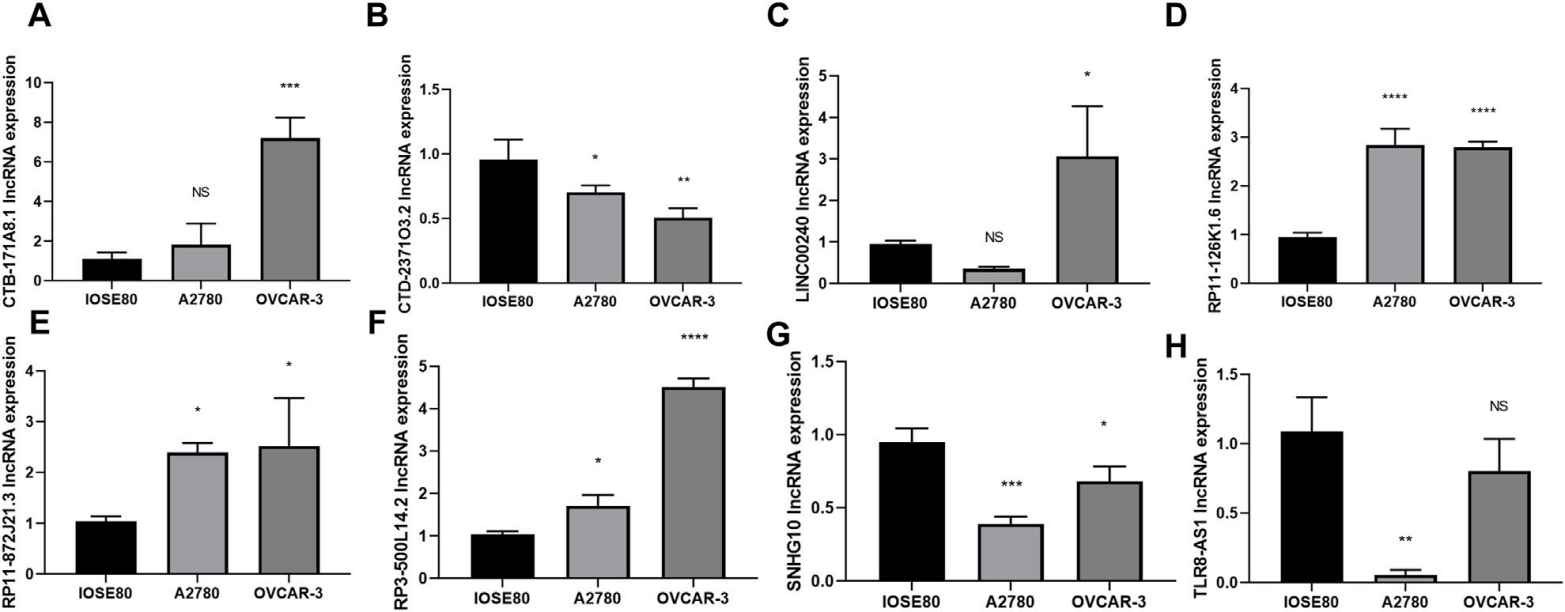
Evaluation of the 8 core DRLs at the cellular level **(A–H)** The expression of CTB-171A8.1, CTD-2371O3.2, LINC00240, RP11-126K1.6, RP11-872J21.3, RP3-500L14.2, SNHG10 and TLR8-AS1 in human ovarian cancer cell lines (A2780 and OVCAR-3) and a normal ovarian cell line (IOSE80). *p < 0.05; **p < 0.01; ***p < 0.001.

To further investigate the expression of the 8 core DRLs cluster at the cellular level, we conducted qRT-PCR analysis to measure the expression levels of the cluster in human ovarian cancer cell lines (A2780 and OVCAR-3) and a normal ovarian cell line (IOSE80). Our results revealed that CTB-171A8.1, RP11-126K1.6, RP11-872J21.3, and RP3-500L14.2 were upregulated in the human ovarian cancer cell lines (A2780 and OVCAR-3), consistent with our model results. Conversely, CTD-2371O3.2 and SNHG10 showed downregulated expression, also in accordance with the model results. LINC00240 exhibited varying expression patterns in human ovarian cancer cell lines A2780 and OVCAR-3, while TRL8 was found to be downregulated in these cell lines, contrary to the model predictions. These inconsistencies could be due to the small sample size in our study, potentially introducing bias (Figures 3A–H).

### Evaluation of the 8 core DRLs at the single-cell RNA sequencing level

**FIGURE 4:**
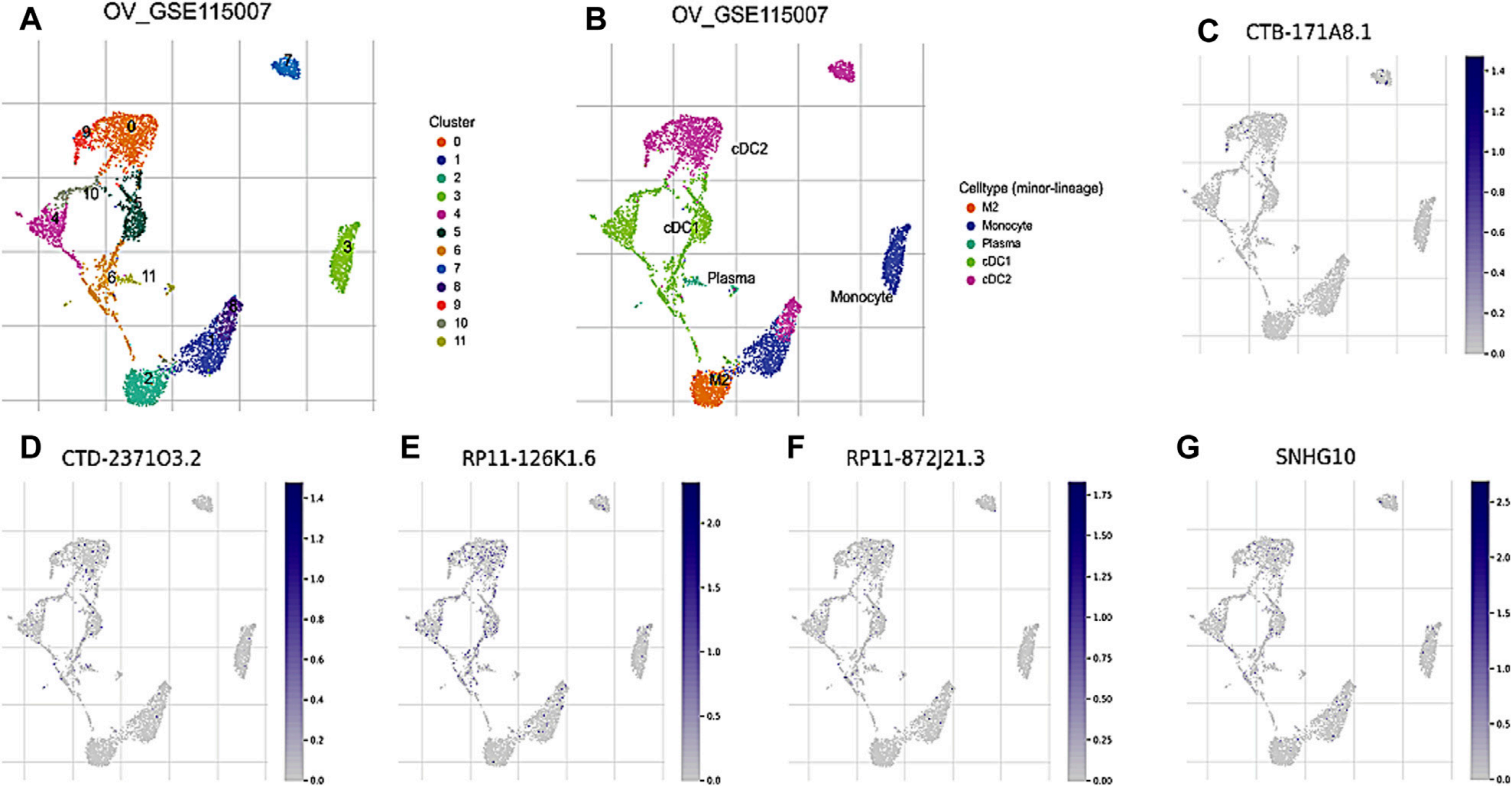
Evaluation of the 8 core DRLs at the scRNA-seq level **(A)** The identified cell clusters in ovarian cancer tissues based on the GSE115007 dataset. **(B)** The identified cell types in ovarian cancer tissues based on the GSE115007 dataset. **(C–G)** The expression levels of CTB-171A8.1, CTD-2371O3.2, RP11-126K1.6, RP11-872J21.3, and SNHG10 in the identified cell types in ovarian cancer tissues based on the GSE115007 dataset.

To further investigate the prognostic values and unique distribution of the 8 core DRLs cluster, our study aimed to explore the specific cell types in which they are enriched using scRNA-seq. Subsequently, we identified the expression of 5 lncRNAs (CTB-171A8.1, CTD-2371O3.2, RP11-126K1.6, RP11-872J21.3, and SNHG10) at the single-cell sequencing level. Analysis of the scRNA-seq data in the GSE115007 dataset showed that 12 cell clusters and 5 cell types are identified in CRC tissues (Figure 4A, B). Our analysis revealed that CTB-171A8.1 is notably enriched in the cDC2, while CTD-2371O3.2 shows significant enrichment in both cDC1, cDC2, and plasma cells. Additionally, RP11-126K1.6 exhibits significant enrichment in cDC1, cDC2, and monocytes, whereas RP11-872J21.3 is notably enriched in cDC1 and cDC2 subsets. Furthermore, SNHG10 displays significant enrichment in cDC1, cDC2, and monocytes (Figure 4C-G).

### Prognosis prediction abilities of the core DRLs

**FIGURE 5:**
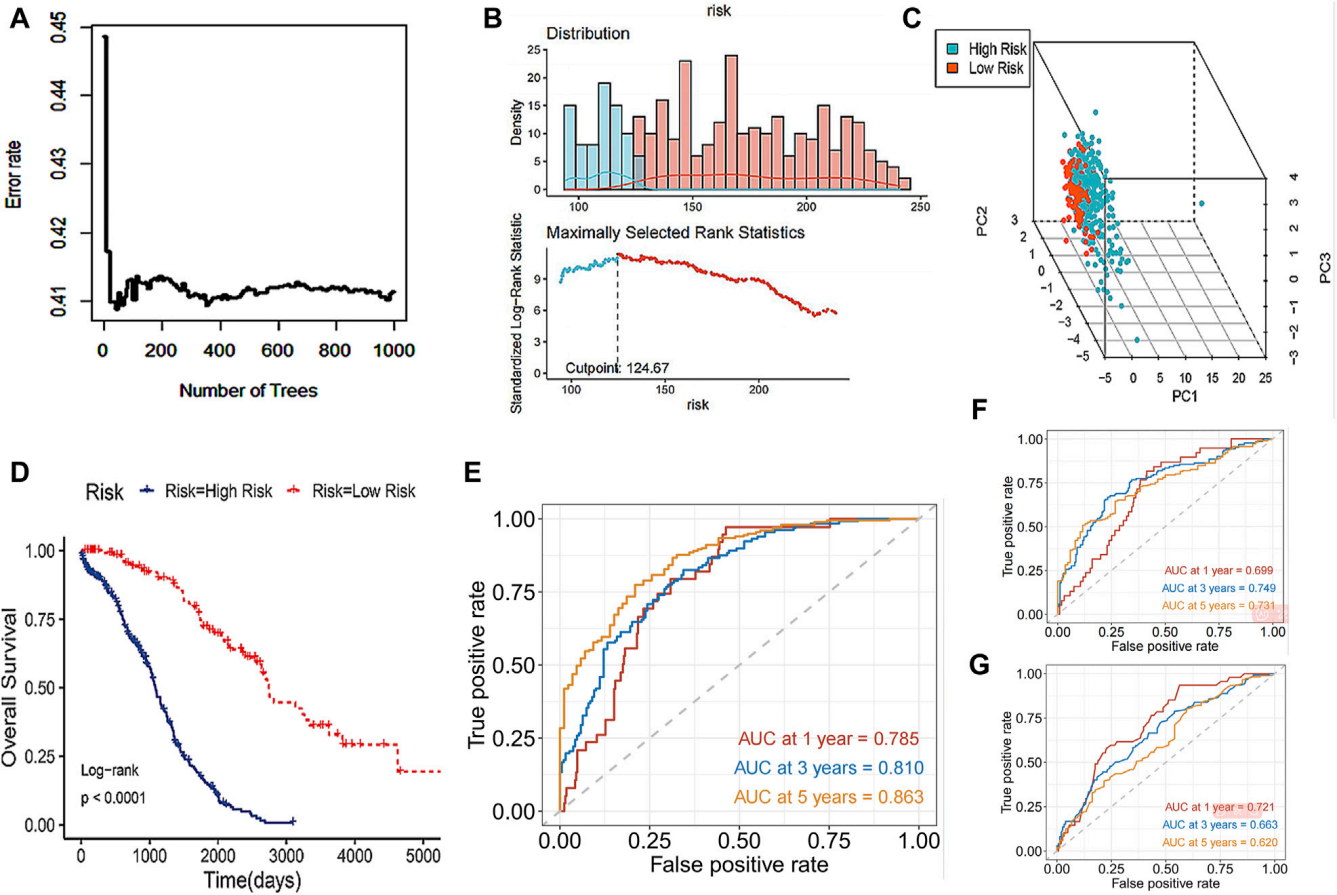
Prognosis prediction abilities of the core DRLs. **(A)** RSF, a machine learning algorithm, was utilized to construct a prognostic model for ovarian cancer patients. **(B)** The surv_cutpoint function from the R package was utilized to determine the optimal cutoff value for 8 core DRLs in predicting the prognostic risk of ovarian cancer. Then patients were divided into low-risk and high-risk groups based on this threshold. Blue represents the low-risk group and red represents the high-risk group. **(C)** PCA analysis showed the prognostic model had a good discrimination in two groups. **(D)** KM curve of overall survival (OS) for ovarian cancer patients in the high-risk and low-risk groups. **(E)** In the RSF prediction model, the AUCs of 1-year, 3-year and 5-year OS in the TCGA cohort were 0.785, 0.810 and 0.863 respectively. **(F)** In the LASSO regression prediction model, the AUC values for 1-year, 3-year, and 5-year OS in the TCGA cohort were 0.699, 0.749, and 0.731, respectively. **(G)** In the clinical features prediction model, the AUC values for 1-year, 3-year, and 5-year OS in the TCGA cohort were 0.721, 0.633, and 0.620, respectively.

The study incorporated the 8 core DRLs into the Random Survival Forest (RSF) to develop a prognostic model, as illustrated in Figure 5A, and the model achieved a C-index of 0.753 with a 95% confidence interval of 0.672–0.820. Subsequently, individual risk scores were computed for each patient. In this study, we utilized the surv_cutpoint function from the R package to determine the optimal cutoff value for 8 core DRLs in predicting the prognostic risk of ovarian cancer. Then patients were divided into low-risk and high-risk groups based on this threshold (Figure 5B). Notably, the high-risk group comprised 297 patients, while the low-risk group consisted of 121 patients. As shown in Figure 5C, PCA illustrated the model’s effective discriminatory capabilities between these two groups. Survival analysis revealed a significant reduction in overall survival (OS) within the high-risk group (Figure 5D). Our study compared the testing performance of three distinct ovarian cancer prognosis prediction models developed using the RSF, LASSO regression, and other clinical features including age, tumor grade, pathological type, and tumor stage. The findings revealed that in the RSF prediction model, the AUCs for 1-year, 3-year, and 5-year OS in the TCGA cohort were 0.785, 0.810, and 0.863, respectively (Figure 5E). For the LASSO regression prediction model, the AUC values for 1-year, 3-year, and 5-year OS in the TCGA cohort were 0.699, 0.749, and 0.731, respectively (Figure 5F). Lastly, in the clinical features prediction model, the AUC values for 1-year, 3-year, and 5-year OS in the TCGA cohort were 0.721, 0.633, and 0.620, respectively (Figure 5G). Our results indicated that the RSF prediction model demonstrated superior prediction performance for prognostic evaluation of disulfidptosis-related ovarian cancer compared to the other two methods in this study.

## Functional enrichment analysis

**FIGURE 6:**
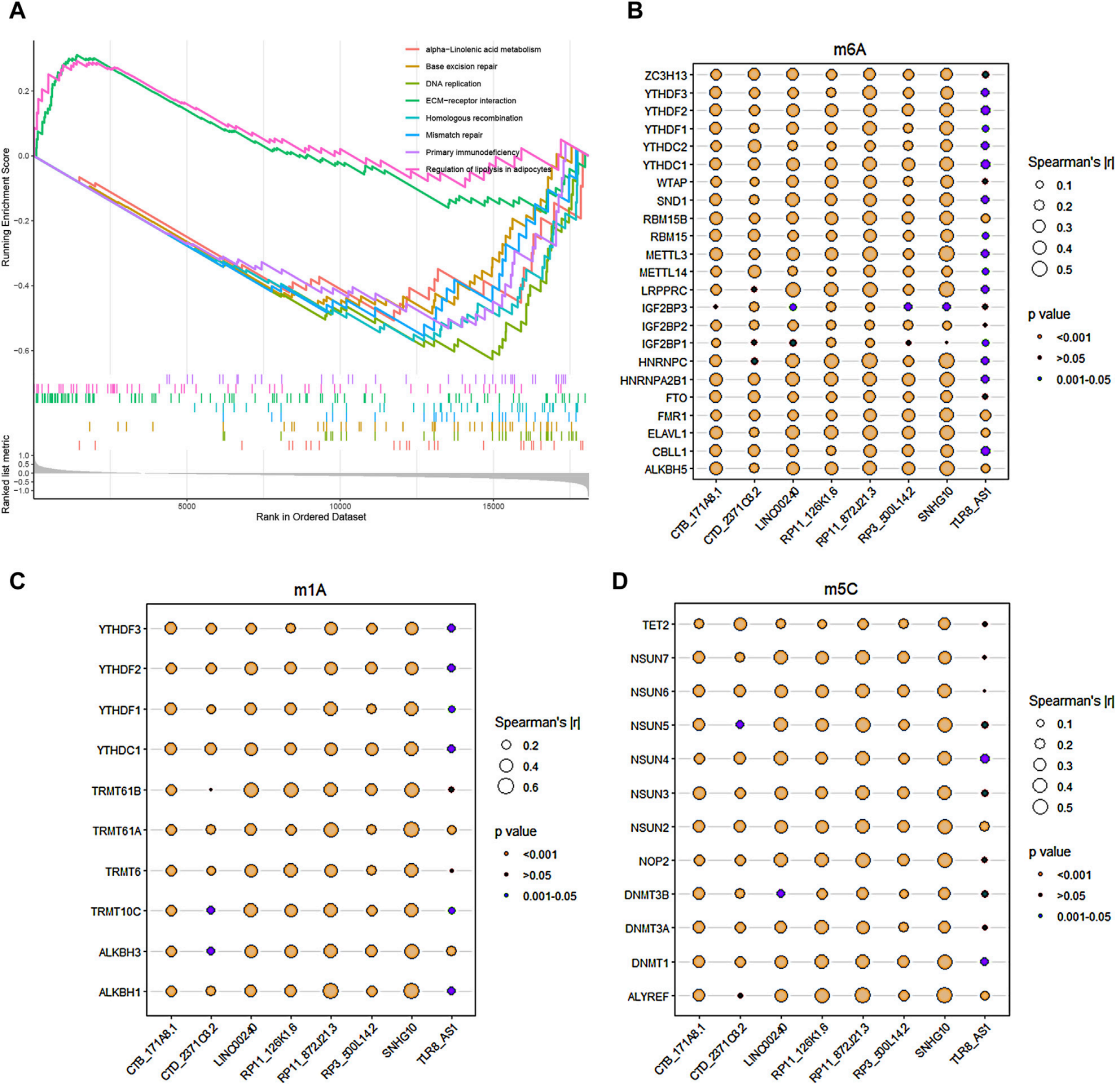
Functional enrichment analysis and RNA methylation. **(A)** GSEA enrichment analysis revealed the biological functions and pathways in the high-risk and low-risk groups. **(B–D)** The correlations between the 8 core DRLs and RNA methylation genes, including m6A modification-related genes **(B)**, m1A modification-related genes **(C)** and m5C modification-related genes **(D)**, respectively.

The GSEA functional enrichment analysis identified significant enrichment of pathways related to ‘ECM-receptor interaction’ and ‘regulation of lipolysis in adipocytes’ in the high-risk group. Conversely, the low-risk group exhibited enrichment in pathways including ‘base excision repair’, ‘alpha-Linolenic acid metabolism’, ‘primary immunodeficiency’, ‘mismatch repair’, ‘homologous recombination’, and ‘DNA replication’ (Figure 6A).

### RNA methylation of the core DRLs

The study utilized Pearson analysis to examine the associations between the 8 core DRLs and RNA methylation genes. Bubble charts were employed to visually represent these correlations, with bubble size indicating the strength of the relationship. Larger bubbles denote stronger correlations between DRLs and RNA methylation genes. The significance of the correlation is depicted by different bubble colors, with brown and dark-cyan indicating statistical significance. In Figures 5B–D, the results demonstrated robust correlations (|r| > 0.5) between LINC00240, RP11-126K1.6, RP11-872J21.3, and SNHG10 with RNA m6A, m1A, and m5C modification genes, suggesting potential links between disulfidptosis and RNA methylation. The methylation levels of disulfidptosis regulators may play a role in tumor progression.

### Immune landscapes analysis

**FIGURE 7:**
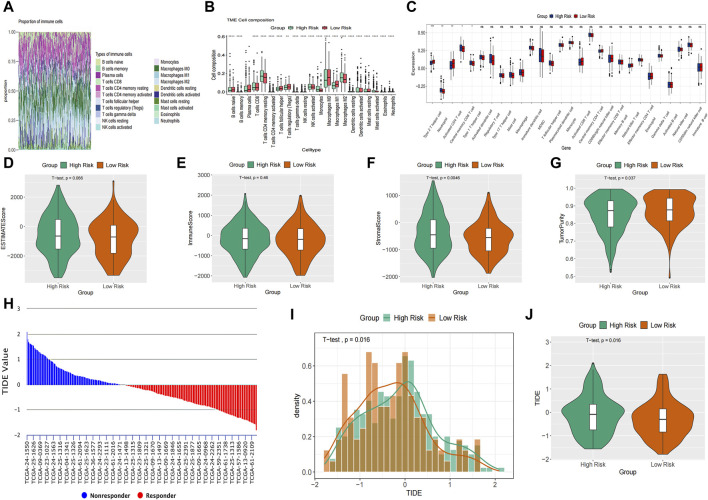
Immune landscapes analysis. **(A)** The composition percentages of 22 immune cell types in each sample. **(B)** The box plot illustrated the differences calculated by CIBERSORT in 22 types of immune infiltration cells between the high-risk and low-risk groups. **(C)** Comparison of the ssGSEA scores of immune cells between high-risk and low-risk groups. The statistical differences were shown as follow: ns, not significant; *p < 0.05; **p < 0.01; ***p < 0.001. **(D–G)** The violin plots showed the differences between high-risk and low-risk groups inestimated score **(D)**, immune score **(E)**, stromal score **(F)** and tumor purity **(G)** calculated using the ESTIMATE algorithm. **(H–J)** Differences in TIDE between the high-risk and low risk groups. The histogram showed the TIDE score of each sample **(H)** and distribution density between two groups **(I)**. The violin plots manifested the differences of TIDE score between two groups **(J)**.

The study utilized CIBERSORT to analyze the composition percentages of 22 immune cell types in each sample, visualizing the results in a heat map (Figure 7A). Additionally, a box plot was employed to demonstrate differences in immune cell infiltration in the tumor microenvironment (TME) between the high-risk and low-risk groups (Figure 7B). The high-risk group exhibited higher expressions of T cells CD4 memory resting, monocytes, and macrophages M2, whereas the low-risk group showed higher proportions of T cells follicular helper, T cells regulatory, and macrophages M0/1. The results of ssGSEA indicated elevated neutrophil and type 1 T helper cell expressions in the high-risk group compared to the low-risk group (Figure 7C). Furthermore, ESTIMATE analysis revealed a higher stromal score in the high-risk group and increased tumor purity in the low-risk group (Figures 7D–G). Previous studies have indicated that low tumor purity is linked to unfavorable prognosis and immune evasion characteristics (Gong et al., 2020; Zhang et al., 2017). These findings indicate notable differences in immune function between the two risk groups, which could potentially affect survival outcomes. The TIDE score was significantly higher in the high-risk group, suggesting a higher probability of tumor cells evading immune surveillance and showing a limited response to immunotherapy (Figures 7H–J).

### TMB analysis

**FIGURE 8:**
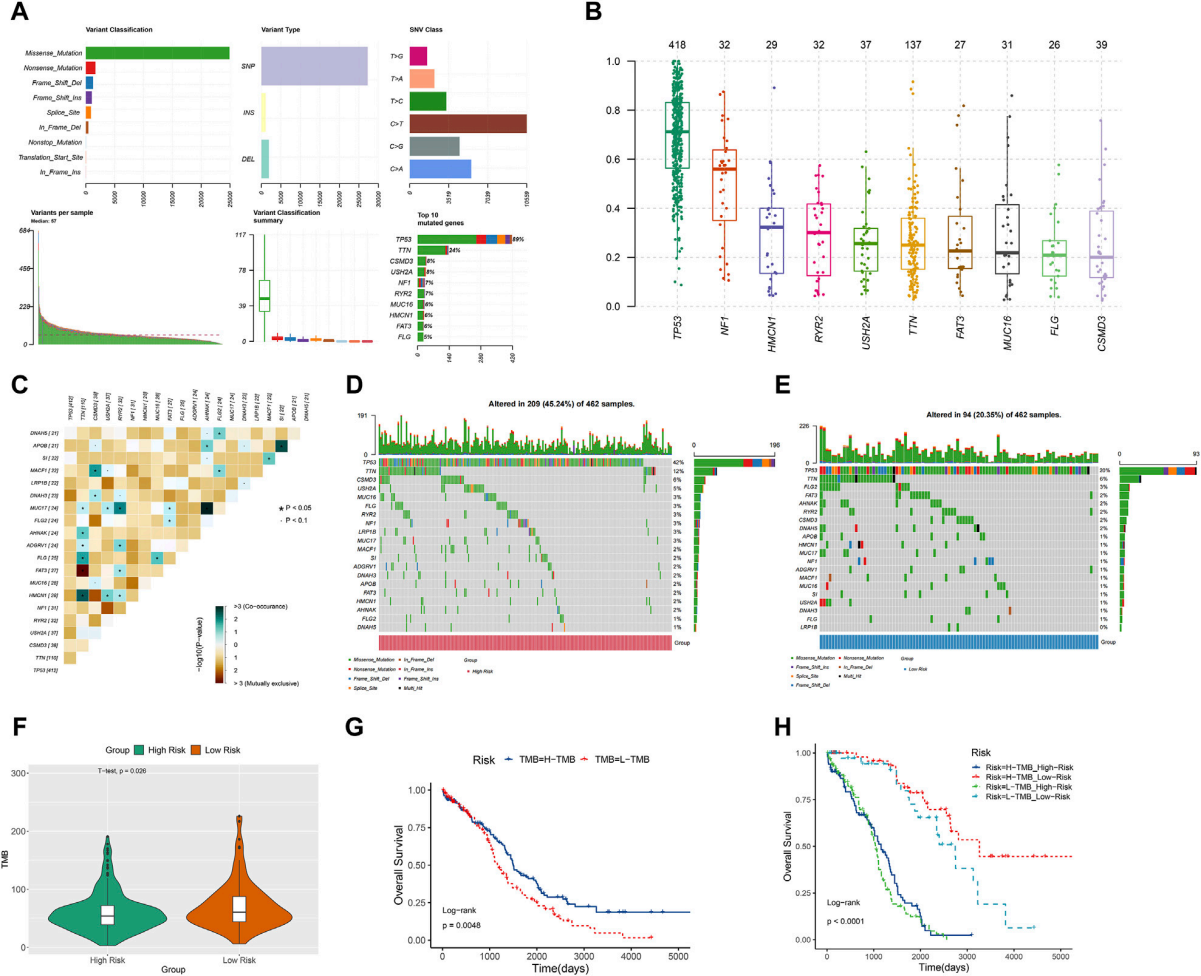
TMB analysis. **(A)** The Cohort summary plot showed the variant classification, type, and SNV Class. The lower part describes the mutation load and variant classification type for each sample. The stacked bar plot diaplayed the top 10 mutated genes. **(B)** The variant allele frequencies (VAF) box plot exhibited the cloning status of the gene. **(D)** The waterfall plot displayed the mutation information of each gene within high-risk group. **(C)** The heatmap manifested the genetic mutual exclusion or co-occurrence. **(E)** The waterfall plot displayed the mutation information of each gene within low-risk group. **(F)** The violin plot showed the differences of somatic mutations between the high-risk and low-risk groups. **(G)** KM curve of OS for ovarian cancer patients in the high-TMB and low-TMB groups. **(H)** The relation among OS, risk scores and TMB in ovarian cancer.

TMB, a measure of mutations associated with T cell recognition, has potential as a prognostic factor in anti-tumor immunotherapy (Liu S. et al., 2023). In Figure 8A, missense mutations were predominant, with C>T point mutations being most common, particularly in TP53 and TTN genes. The gene’s variant allele frequencies (VAF) box plot illustrated gene cloning status (Figure 8B). The somaticInteractions function in R was utilized to analyze genetic mutations for mutual exclusion or co-occurrence (Figure 8C). Waterfall charts depicted somatic mutation status in high-risk and low-risk groups (Figures 8D, E). Notably, TP53, TTN, and CSMD3 were the top 3 genes with highest mutation probability in the high-risk group, while TP53, TTN, and FLG2 showed highest mutation probability in the low-risk group. Violin plots indicated a lower somatic mutation rate in the high-risk group compared to the low-risk group (Figure 8F). Furthermore, a survival rate difference was observed between patients with high and low TMB (Figure 8G). Utilizing TMB and risk scores for predicting ovarian cancer patients’ prognosis provided a more comprehensive and precise predictive effect (Figure 8H).

### Drug sensitivity analysis

A sensitivity analysis of chemotherapy drugs was carried out using the GDSC database to compare the half-maximal inhibitory concentration (IC50) values between high-risk and low-risk groups. The study revealed that bleomycin had a lower IC50 value in the high-risk group, whereas GSK1904529A, vorinostat, phenformin, ZM-447439, rapamycin, OSI-930, NG-25, vinorelbine, and EHT1864 exhibited higher IC50 values in the high-risk group. Conversely, cisplatin, paclitaxel, and docetaxel did not exhibit a statistically significant difference in IC50 values between the low- and high-risk groups (Supplementary Figure S1A-M). These findings may provide new insights into the treatment of ovarian cancer, but further validation is needed.

## Discussion

Ovarian cancer has the highest mortality rate among gynecological cancers in women. The standard treatment for ovarian cancer is standardized surgical staging, followed by postoperative systemic platinum-based chemotherapy and/or targeted therapy (Konstantinopoulos and Matulonis, 2023; Salutari et al., 2024). Advanced stage at diagnosis and platinum-resistant recurrence are the primary factors contributing to the poor prognosis of ovarian cancer (Pavlik et al., 2024). There is an urgent need to identify new targets, improve early detection and prediction, and develop innovative treatments for ovarian cancer. Prior studies by Liu and Wang et al. (Liu L. et al., 2023) constructed a prognostic signature using cuproptosis-related lncRNAs for ovarian cancer patients and the AUC values for the testing dataset at 3 and 5 years were reported as 0.627 and 0.633, respectively. Additionally, Xu et al. (Xu et al., 2023). established a prognostic signature based on pyroptosis-related lncRNAs for ovarian cancer and the survival rates at 1-year, 3-year and 5-year were found to be 0.688, 0.703 and 0.742, respectively. In our study, we identified 7,174 DELs between ovarian cancer patients and normal samples, and 2,467 DRLs were identified using Pearson correlation analysis. The 8 core DRLs were selected through LASSO regression. RSF was utilized to develop an ovarian cancer prognostic model. The AUCs for 1-year, 3-year, and 5-year OS in the TCGA cohort were 0.785, 0.810, and 0.863, respectively. Meanwhile, the testing performance of the prognostic prediction model for disulfidptosis-related ovarian cancer was compared among RSF, LASSO regression, and other clinical information. The findings indicated that the prognostic prediction model developed using RSF demonstrated superior testing performance. These results suggest that the 8 core DRLs may serve as potential biomarkers for identifying the prognosis of ovarian cancer patients.

Alterations in metabolic processes are a distinguishing characteristic of cancer, providing potential targets for precise intervention in cancer treatment (Zheng et al., 2023). Disulfidptosis, a novel form of cell death, has been identified as a significant factor in the development of various tumors, opening up new possibilities for tumor treatment (Liu et al., 2023f). Research has indicated that disulfidptosis-related metabolism plays a significant role in tumor cell metastasis, drug resistance, and immune evasion (Xie et al., 2024). Tang et al. (Tang et al., 2024) identified five key genes and emphasized the significance of a disulfidptosis-related gene signature in predicting breast cancer prognosis. Pu et al. (Pu et al., 2024) utilized machine learning methods to establish a disulfidptosis-associated lncRNA signature for forecasting the prognosis and immune response in hepatocellular carcinoma, yielding promising predictive outcomes. There is currently a lack of analysis on the correlation between disulfidptosis and ovarian cancer from the perspective of lncRNA. Our study aims to address this gap in research. This study is the first to create a new prognostic risk model for ovarian cancer using the 8 core DRLs, and investigate the impact of disulfidptosis on ovarian cancer from various angles including immune infiltration and methylation. The findings offer valuable insights for immunotherapy, chemotherapy, and can assist in predicting prognosis for ovarian cancer. In this study, ovarian cancer patients were divided into low-risk and high-risk groups, with high-risk samples exhibiting significantly lower OS compared to low-risk samples. Furthermore, differences in the immune landscape between high-risk and low-risk groups were analyzed. The study also identified variances in immune cell infiltration types, activity, and somatic mutation status between the two groups. Tumor development and progression involve intricate interactions among cancer cells, the immune system, and the tumor microenvironment (TME). These factors regulate the strength and duration of the anti-cancer response (Chen and Mellman, 2017). Prior research has indicated that the TME of ovarian cancer is intricate and dynamic, playing a crucial role in advancing tumor growth, invasion, and resistance to chemotherapy. Immune cells and components within the TME have a dual role in both restraining tumor growth and facilitating immune evasion. These elements maintain a delicate balance and are vital for processes like extracellular matrix remodeling, the activation of cancer-related fibroblasts, and metabolic reprogramming (Blanc-Durand et al., 2023). RNA methylation is known to play crucial roles in cancer development (Xia et al., 2023). The most common internal mRNA modification in eukaryotic cells is RNA m6A modification, which regulates various RNA processing steps. On the other hand, RNA m1A modification disrupts base pairing and has the potential to impact local RNA structure or protein-RNA interactions (Wang et al., 2017). In our study, we investigated the correlations between 8 core DRLs and RNA methylation genes, uncovering potential links between disulfidptosis and RNA methylation. While chemotherapy remains a key strategy for treating ovarian cancer, issues such as tumor heterogeneity and drug resistance often result in reduced chemotherapy efficacy (Wang et al., 2024). Chemotherapy resistance contributes significantly to the high mortality rate of ovarian cancer (Atallah et al., 2023). In this study, the different responses of patients in high-risk and low-risk groups to drugs indicate that tailoring treatments based on patient risk groups may lead to more effective outcomes. Combining the sensitivity of tumor cells to disulfidptosis with the anti-cancer effects of other drugs may offer new insights for the development of innovative and potent cancer therapies, but further experimental validation is necessary. Studies have reported that disulfidptosis is associated with immune-related characteristics, and patients with high disulfidptosis activity have a better prognosis after immunotherapy compared to patients with low disulfidptosis activity (Xie et al., 2024). In addition, qRT-PCR was utilized to analyze the expression of 8 core DRLs in human ovarian cancer cell lines and normal ovarian tissues at the cellular level. Previous research has indicated that decreased expression of SNHG10 is correlated with a poor prognosis in ovarian cancer patients. The overexpression of SNHG10 has been shown to suppress the proliferation, colony formation, migration, and invasion of ovarian cancer cells (Lv et al., 2022). Further exploration was conducted on the expression of the 5 DRLs at the scRNA-seq levels. Our findings indicated that these DRLs are predominantly enriched in DC cells, plasma cells, and monocytes.

Although the predictive performance of the disulfidptosis-related lncRNAs cluster in forecasting the prognosis and immune landscapes of ovarian cancer has been validated, there are still some limitations. Firstly, The data were sourced from a single database, but additional data from large-scale multicenter cohorts are needed to assess the predictive signatures effectively. Secondly, further experiments are required to validate the findings and clarify the involvement of DRLs in ovarian cancer. It is necessary to delve deeper into the mechanisms underlying the role of the 8 core DRLs in ovarian cancer. Thirdly, scRNA-seq expression data for 5 lncRNAs (CTB-171A8.1, CTD-2371O3.2, RP11-126K1.6, RP11-872J21.3, and SNHG10) was identified in the TISCH database. However, additional research and exploration is required for the other 3 lncRNAs (LINC00240, RP3-500L14.2, and TLR8-AS1) in the future.

## Conclusion

Our study developed a prediction model for a cluster of disulfidptosis-related lncRNAs to predict the prognosis and immune landscapes of ovarian cancer, yielding positive outcomes. Building upon prior research findings, we hypothesized that uncovering the prognostic and therapeutic implications of lncRNAs associated with disulfidptosis could enhance the assessment and management of ovarian cancer in patients.

## Data Availability

The codes for analysis and data are available online at https://github.com/weijiahui11/disulfidptosis-lncRNA_ovarian-cancer.
