# Supplementary material for: A disulfidptosis-related lncRNAs cluster to forecast the prognosis and immune landscapes of ovarian cancer
Source: Front Genet. 2024 Jul 9;15:1397011. doi: 10.3389/fgene.2024.1397011 (PMC11263023; doi:10.3389/fgene.2024.1397011)
Supplement: Supplementary file 1 [file Table1.docx]

Supplementary table 1 the 8 DRLs cluster SYBR primer comparison table

| LncRNA name | Primer Sequence |
| --- | --- |
| CTB-171A8.1 | 5′-GCACATGATGACATCTGGCAAT-3′  5′-CAGGTTTGCTTCGCTTTATGTA-3′ |
| CTD-2371O3.2 | 5′-CGAGGACTAAATGAGGCAAGG-3′  5′-GGTGCGGTTGGGATTTATATATG-3′ |
| LINC00240 | 5ʹ-TCGTGTAGTTTGTTCCACCCTG-3ʹ  5ʹ-GGAAGCAGTGAAGGTAGATGTCA-3ʹ |
| RP11-126K1.6 | 5ʹ-AAGGGACCGCAGGATGAAA-3ʹ  5ʹ-AGCTCTGAGGACCGTAGACTAAAC-3ʹ |
| RP11-872J21.3 | 5ʹ-TGTATTCCTGATGCCTGACCC-3ʹ  5ʹ-GTGACTGCCATTGATGTTCCTAT-3ʹ |
| RP3-500L14.2 | 5ʹ-AAGGGGTGGCATCTGGTGA-3ʹ  5ʹ-GCTCTGCCCTTGTGAGTGGAT-3ʹ |
| SNHG10 | 5ʹ-GTCTCGAACTCCTGGCTTCAA-3ʹ  5ʹ-TCTCATTTCTCACGATGGGTCC-3ʹ |
| TLR8-AS1-S | 5ʹ-TTGTTCATATCGTGGAACTCTTAGA-3ʹ  5ʹ-GTGAAGTCTGTAACAACAAGCGTAG-3ʹ |
| GAPDH | 5ʹ-GGAAGCTTGTCATCAATGGAAATC-3ʹ  5ʹ-TGATGACCCTTTTGGCTCCC-3ʹ |
